# Leukocyte telomere length is inversely associated with arterial wave reflection in 566 normotensive and never-treated hypertensive subjects

**DOI:** 10.18632/aging.103459

**Published:** 2020-06-23

**Authors:** Milja Honkonen, Kati Vääräniemi, Outi Saijonmaa, Anna Nyman, Antti J. Tikkakoski, Jenni Koskela, Terho Lehtimäki, Mika Kähönen, Jukka Mustonen, Frej Fyhrquist, Ilkka Pörsti

**Affiliations:** 1 Faculty of Medicine and Health Technology and Finnish Cardiovascular Research Center Tampere, Tampere University, Tampere, Finland; 2 Department of Internal Medicine, Central Hospital of Central Finland, Jyväskylä, Finland; 3 Minerva Institute for Medical Research, Biomedicum U2 Helsinki, Helsinki, Finland; 4 Department of Clinical Physiology, Tampere University Hospital, Tampere, Finland; 5 Department of Internal Medicine, Tampere University Hospital, Tampere, Finland; 6 Department of Clinical Chemistry, Fimlab Laboratories Ltd, Tampere, Finland

**Keywords:** aging, circulatory system, hemodynamics, telomeres

## Abstract

Telomeres are short segments in chromosome ends, the length of which is reduced during cell lifecycles. We examined the association of mean leukocyte telomere length (LTL) and short telomere proportion (STP) with hemodynamic variables in normotensive and never-treated hypertensive volunteers (n=566, 19-72 years). STP and mean LTL were determined using Southern blotting, and supine hemodynamics recorded using continuous tonometric pulse wave analysis and whole-body impedance cardiography. The analyses were adjusted for age, body mass index (BMI), alcohol use, smoking, plasma chemistry, and estimated glomerular filtration rate (eGFR). In univariate analyses, mean LTL and STP both correlated with age, BMI, eGFR, aortic blood pressure, augmentation index, and pulse wave velocity (p<0.05 for all). Mean LTL also correlated with systemic vascular resistance (p<0.05). In linear regression analyses of all hemodynamic variables, mean LTL was only an independent explanatory factor for augmentation index (Beta -0.006, p=0.032), while STP was not an explanatory factor for any of the hemodynamic variables, in contrast to age, BMI and several cardiovascular risk factors. To conclude, augmentation index was predominantly related with chronological aging, but also with mean LTL, suggesting that this variable of central wave reflection is a modest marker of vascular biological aging.

## INTRODUCTION

Telomeres have several important functions for cell homeostasis, such as preventing chromosomes from attaching to each other and helping deoxyribonucleic acid (DNA) repairing to function properly. Without telomeres, DNA repair mechanisms would misrecognize chromosomes as broken DNA, and this would lead to inappropriately connected chromosomes [1]. Telomere shortening takes place during every life cycle of cells due to the lagging DNA strand that cannot be completely replicated because of the Okazaki fragments [2]. Short telomeres have a more pronounced influence on cell senescence than long telomeres [3], and when the telomeres become critically short, the cell enters replicative senescence and finally apoptosis [4].

Reduced leukocyte telomere length (LTL) has been associated with cardiovascular risk factors, such as family history of cardiovascular disease, lower endothelial progenitor cell number, and lower high-density lipoprotein cholesterol (HDL-C) concentration [5]. In addition to aging, lifestyle factors like smoking and alcohol consumption, and several cardiovascular risk factors may accelerate endothelial cell senescence via telomere shortening and damage due to oxidative stress and inflammation [6, 7]. Increased cell turnover and higher hemodynamic stress may wear telomeres down, and shortening of telomeres has been found to be more apparent in the arterial intima than in the media [8].

Shorter LTL was associated with impaired pressure-diameter relation of the carotid artery in American Indians [9], while men but not women with shorter LTL were more likely to have increased pulse pressure and pulse wave velocity (PWV) [10]. These findings associate telomere shortening with arterial stiffness. The characteristic aging-related changes in the cardiovascular system are increased large arterial stiffness with subsequent increases in systolic blood pressure (BP) and pulse pressure, and enhanced arterial wave reflections, manifested as higher level of augmentation index (AIx) [11–13].

In 163 hypertensive men treated with BP and lipid lowering medications, shorter LTL was related to the presence of carotid artery plaques [14]. LTL was also shorter in 203 patients with premature myocardial infarction (<50 years of age) than in 180 healthy controls [15]. Patients with coronary heart disease induced cardiac failure had shorter LTL than healthy subjects of the same age [16]. Also 190 patients with abdominal aortic aneurysm had shorter LTL when compared with 183 controls, suggesting a role for telomeres in vascular biological aging [17]. Reduced LTL has even been reported to moderately predict cardiovascular disease in drug-treated patients with hypertension and left ventricular hypertrophy [18]. In contrast, Denil et al. examined 2524 subjects free from cardiovascular disease, aged 35-55 years, and found no correlations between LTL and PWV, pulse pressure, left ventricular mass index, ejection fraction, and peak systolic septal annular motion [19]. However, LTL was associated with variables reflecting left ventricular filling [19].

According to a recent review, we do not have the final evidence whether telomere shortening is a true cause or merely a consequence of cardiovascular disease [20]. Altogether, detailed information about the association of LTL with hemodynamic variables is scarce, especially in subjects not using antihypertensive medications or other compounds with direct influences on cardiovascular function. Here we tested the hypothesis whether mean LTL or short telomere proportion (STP), determined using Southern blotting, were independently associated with hemodynamic variables in 566 normotensive and never-treated hypertensive volunteers.

## RESULTS

### Study population

The study included 566 subjects, 287 men and 279 women, and their mean age was 45 years (age range 19-72 years) (Table 1). In women, body mass index (BMI), use of alcohol, proportion of present and previous smokers, and office BP were lower than in men. The mean LTL was higher, while STP was lower, in women than in men (Table 1). Women also presented with lower blood hemoglobin, plasma potassium, uric acid, glucose, and more favorable lipid profile than men. Although plasma cystatin C and creatinine concentrations were lower in women, estimated glomerular filtration rate (eGFR) calculated from cystatin C plus creatinine concentrations was similar in both sexes (Table 1).

**Table 1:** Characteristics of the study population.

	**All**	**Men**	**Women**
Number	566	287	279
Age (years)	45 (12)	45 (12)	44 (11)
Age range (years)	19-72	19-72	21-72
BMI (kg/m^2^)	26.8 (4.4)	27.4 (3.9)	25.9* (4.7)
Alcohol (standard doses/week)	4.5 (5.8)	6.3 (6.7)	2.7* (3.8)
Smoking (present or previous, %)	29	33	25*
Office blood pressure (mmHg)			
Systolic	141 (21)	146 (20)	135* (20)
Diastolic	89 (13)	92 (12)	86* (12)
Mean telomere length (kb)	8.52 (0.33)	8.49 (0.33)	8.56* (0.32)
Short telomere proportion (%)	11.93 (1.55)	12.07 (1.59)	11.79* (1.50)
Blood hemoglobin (g/l)	144 (12)	152 (8)	136* (9)
eGFR (ml/min per 1.73 m^2^)	98 (15)	98 (15)	99 (14)
Fasting plasma			
Creatinine (μmol/l)	74 (14)	82 (12)	66* (10)
Cystatin C (mg/l)	0.84 (0.15)	0.88 (0.14)	0.80* (0.14)
Sodium (mmol/l)	140 (2)	141 (2)	140 (2)
Potassium (mmol/l)	3.8 (0.3)	3.9 (0.3)	3.8* (0.3)
Uric acid (μmol/l)	302 (77)	348 (67)	255* (55)
Glucose (mmol/l)	5.4 (0.6)	5.6 (0.6)	5.3* (0.5)
CRP (mg/l)	1.6 (2.9)	1.6 (3.6)	1.7 (1.9)
Total cholesterol (mmol/l)	5.1 (1.0)	5.2 (1.1)	5.1 (1.0)
Triglycerides (mmol/l)	1.3 (1.0)	1.5 (1.2)	1.1* (0.6)
HDL cholesterol (mmol/l)	1.6 (0.4)	1.4 (0.3)	1.8* (0.4)
LDL cholesterol (mmol/l)	3.0 (0.96)	3.23 (1.0)	2.8* (0.9)

Mean (standard deviation), *P<0.05 men vs. women; BMI, body mass index; eGFR, cystatin C and creatinine based CDK-EPI formula for estimated glomerular filtration rate [59]; CRP, C-reactive protein; HDL, high density lipoprotein; LDL, low density lipoprotein.

### Hemodynamic measurements

Radial and aortic systolic and diastolic BPs, systemic vascular resistance index (SVRI), and PWV were lower in women than in men (Table 2). Heart rate and the variables of wave reflection, AIx and AIx at heart rate 75/min (AIx@75), were higher in women than in men. Aortic pulse pressure and cardiac index were similar in both sexes (Table 2).

**Table 2:** Hemodynamic variables of the study population.

	**All**	**Men**	**Women**
Radial systolic BP (mmHg)	136 (134-137)	140 (138-142)	131* (128-133)
Radial diastolic BP (mmHg)	79 (78-81)	82 (81-84)	77* (75-78)
Aortic systolic BP (mmHg)	123 (122-125)	126 (124-128)	121* (118-123)
Aortic diastolic BP (mmHg)	80 (79-81)	83 (81-85)	78* (76-79)
Aortic pulse pressure (mmHg)	43 (42-44)	43 (42-44)	43 (42-44)
Heart rate (1/min)	63 (62.7-64.3)	63 (61-64)	65* (63-66)
Cardiac index (l/min per m^2^)	2.92 (2.87-2.96)	2.95 (2.89-3.01)	2.88 (2.83-2.94)
SVRI (dyn*s/cm^5^*m^2^)	2654 (2604-2704)	2721 (2654-2788)	2585* (2513-2657)
Augmentation index (%)	22.6 (21.6-23.6)	18.6 (17.3-19.9)	26.7* (25.4-28.0)
AIx@75 (%)	17.8 (16.8-18.7)	13.3 (12.1-14.6)	22.3* (21.1-23.6)
Pulse wave velocity (m/s)	8.5 (8.3-8.6)	8.9 (8.6-9.1)	8.0* (7.8-8.2)

Mean (95% confidence interval), *P<0.05 men vs. women; BP, blood pressure; AIx, augmentation index; AIx@75, AIx adjusted to heart rate 75/min; SVRI, systemic vascular resistance index.

### Univariate correlations of age, leukocyte telomere length, and short telomere proportion with hemodynamic variables

**Figure 1:**
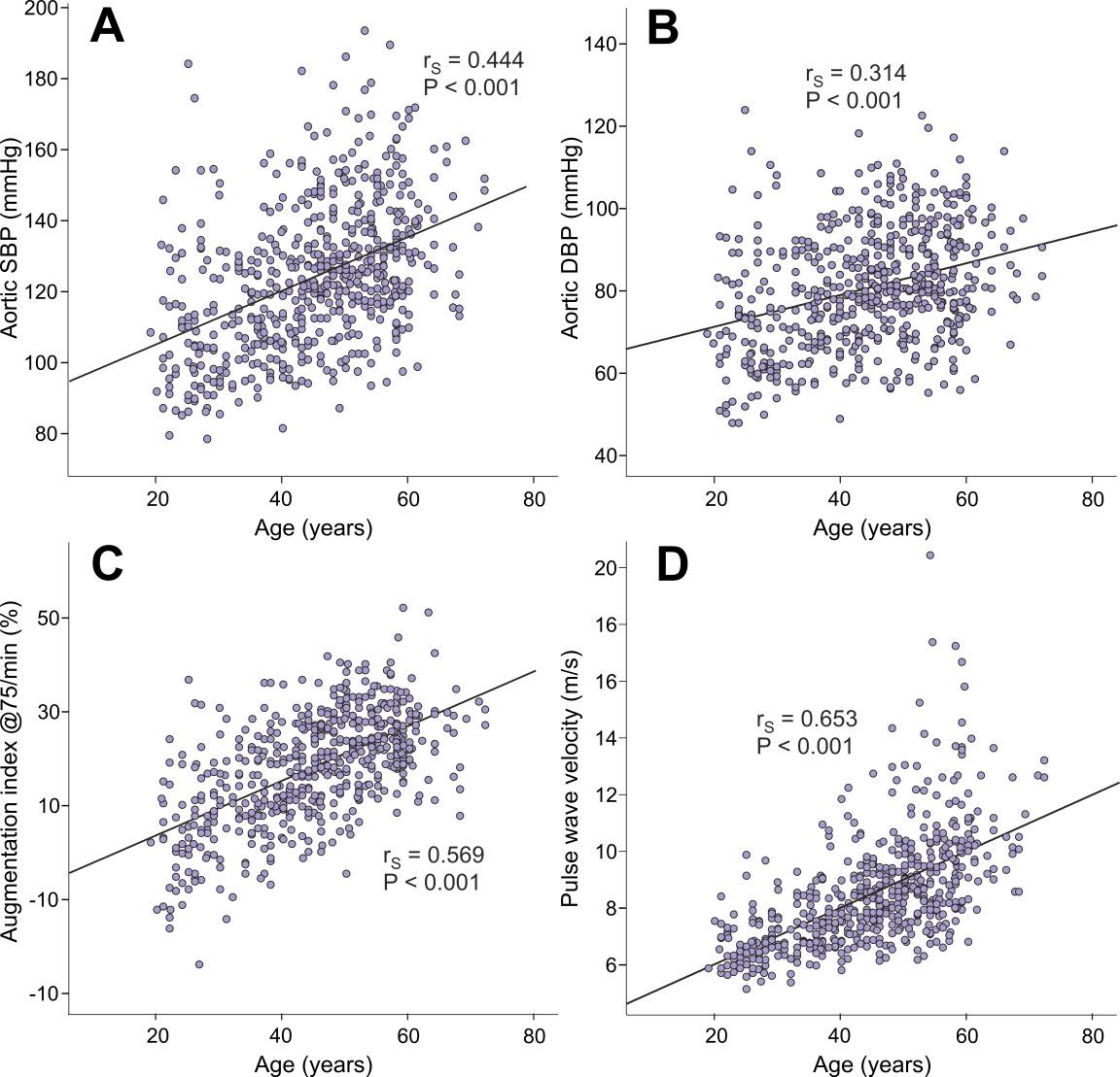
Scatter plots and Spearman correlations (r_S_) between age and aortic systolic blood pressure (**A**), aortic diastolic blood pressure (**B**), augmentation index at heart rate 75/min (**C**), and pulse wave velocity (**D**) in the 566 study subjects.

Age directly correlated with aortic systolic BP (r_S_=0.444), aortic diastolic BP (r_S_=0.314), AIx@75 (r_S_=0.569), and PWV (r_S_=0.653) (Figure 1). Age also correlated with SVRI (r_S_=0.290, p<0.001), but not with heart rate, ventricular ejection duration, or stroke volume (r_S_ range from -0.016 to 0.020, p value range from 0.636 to 0.884). Of note, the variables BP, PWV, systemic vascular resistance, heart rate, ventricular ejection duration, and stroke volume are all hemodynamic determinants of AIx [21–23].

**Figure 2:**
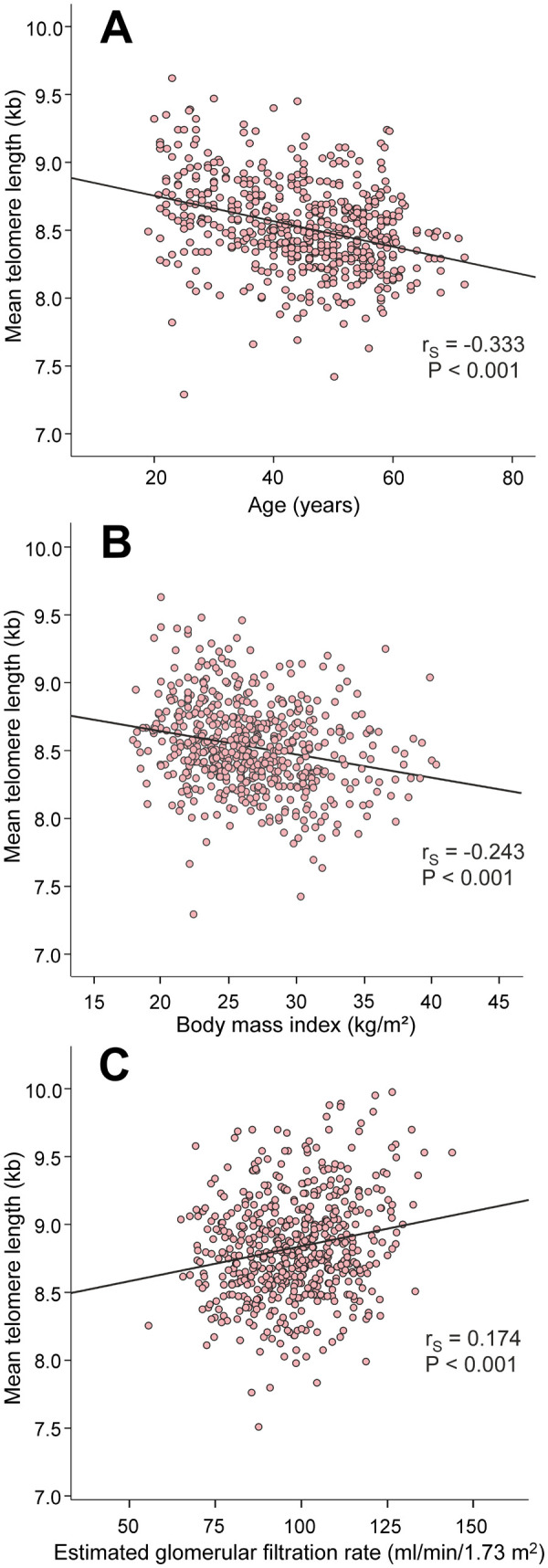
Scatter plots and Spearman correlations (r_S_) between age (**A**), body mass index (**B**), and estimated glomerular filtration rate (**C**), and mean leukocyte telomere length in the 566 study subjects.

The factors that are known to influence LTL presented with expected results: age (r_S_=-0.333) and BMI (r_S_=-0.243) were inversely correlated, while eGFR (r_S_=0.174) was directly correlated with mean LTL (Figure 2).

**Figure 3:**
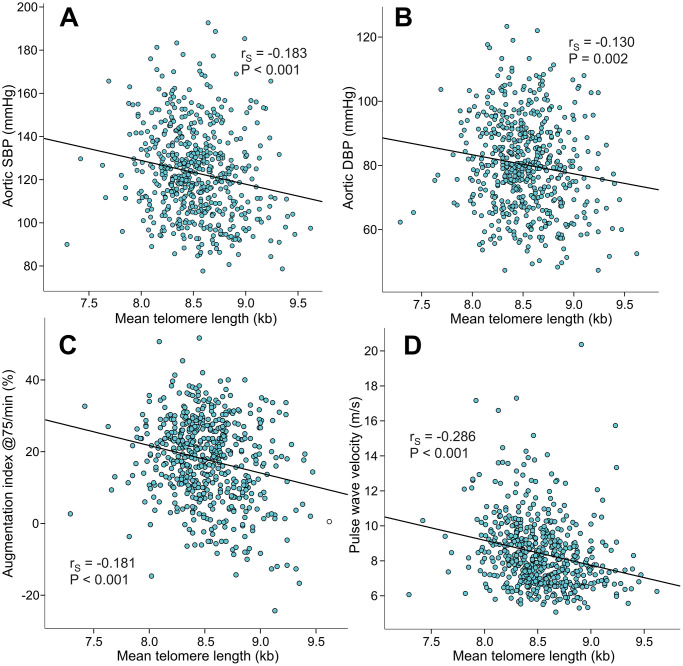
Scatter plots and Spearman correlations (r_S_) between mean leukocyte telomere length and aortic systolic blood pressure (**A**), aortic diastolic blood pressure (**B**), augmentation index at heart rate 75/min (**C**), and pulse wave velocity (**D**) in the 566 study subjects.

Mean LTL inversely correlated with aortic systolic BP (r_S_=-0.183), aortic diastolic BP (r_S_=-0.130), AIx@75 (r_S_=-0.181), PWV (r_S_=-0.286) (Figure 3), and SVRI (r_S_=-0.108, p=0.010). Mean LTL also inversely correlated with the magnitude of AIx (i.e. variable of wave reflection that was not adjusted for heart rate 75/min; r_S_=-0.188, p<0.001, Supplementary Figure 1). Mean LTL did not correlate with heart rate (r_S_=-0.021, p=0.369) or cardiac index (r_S_=-0.025, p=0.547), but was also inversely correlated with office systolic BP (r_S_=-0.167, p<0.001) and office diastolic BP (r_S_=-0.158, p<0.001).

Table 3 shows the correlations of STP with demographic and hemodynamic variables and eGFR. Age (r_S_=0.333), BMI (r_S_=0.151), aortic systolic BP (r_S_=0.073), aortic pulse pressure (r_S_=0.144), AIx (r_S_=0.164), AIx@75 (r_S_=0.161), and PWV (r_S_=0.258) directly correlated, while eGFR inversely correlated (r_S_=-0.141) with STP (Table 3).

**Table 3:** Correlation of short telomere proportion with age, body mass index (BMI), estimated glomerular filtration rate (eGFR), and hemodynamic variables.

**Variable**	**r_S_**	**P-value**	**n**
Age	0.333	<0.001	566
BMI (kg/m^2^)	0.151	0.001	566
eGFR (ml/min/1.73 m^2^)	-0.141	0.001	558
Radial systolic BP (mmHg)	0.073	0.085	565
Radial diastolic BP (mmHg)	0.056	0.182	564
Aortic systolic BP (mmHg)	0.109	0.010	565
Aortic diastolic BP (mmHg)	0.055	0.192	564
Aortic pulse pressure (mmHg)	0.144	0.001	564
Heart rate (1/min)	0.012	0.769	565
Cardiac index (l/min/m^2^)	0.005	0.899	564
SVRI (dyn*s/cm^5^*m^2^)	0.044	0.301	564
Augmentation index (%)	0.164	<0.001	563
AIx@75 (%)	0.161	<0.001	563
Pulse wave velocity (m/s)	0.258	<0.001	564

r_S_, Spearman’s rank correlation; eGFR, cystatin C and creatinine based CDK-EPI formula for eGFR [59]; BP, blood pressure; SVRI, systemic vascular resistance index; AIx@75, augmentation index adjusted to heart rate 75/min.

### Linear regression analyses: explanatory variables for leukocyte telomere length and short telomere proportion

In linear regression analysis with age, sex, BMI, smoking status, categorized alcohol intake, central systolic and diastolic BP, eGFR, and fasting plasma concentrations of potassium, uric acid, low-density lipoprotein cholesterol (LDL-C), and glucose as included variables, the significant explanatory variables for LTL were age (beta -0.293, p<0.001), BMI (beta -0.119, p=0.005), and moderate alcohol consumption category (beta -0.093, p=0.021) (R square value 0.139). The significant explanatory variables for STP were age (beta 0.317, p<0.001), male sex (beta 0.083, p=0.041), and low alcohol intake category (beta -0.117, p=0.004) (R square value 0.123).

### Linear regression analyses: explanatory factors for hemodynamic variables

In addition to mean LTL or STP, the included variables in these analyses were age, sex, BMI, smoking status, categorized alcohol intake, plasma C-reactive protein (CRP), sodium, potassium, uric acid, LDL-C, HDL-C, triglycerides, glucose, and eGFR (Tables 4 and 5). In the analyses of explanatory factors for AIx@75 and AIx, also PWV and SVRI were included in the model. In the analyses of explanatory factors for PWV, aortic systolic BP and heart rate were included in the model.

**Table 4:** Linear regression analyses with backward elimination for the hemodynamic variables including leukocyte telomere length in the explanatory factors.

**Aortic systolic BP (R square 0.337)**				**Aortic diastolic BP (R square 0.290)**			
	**B**	**Beta**	**P**		**B**	**Beta**	**P**
(Constant)	-24.599			(Constant)	77.353		
Age	0.278	0.158	0.001	BMI	0.641	0.196	<0.001
BMI	0.991	0.209	<0.001	High alcohol intake category	10.941	0.117	0.001
High alcohol intake category	13.398	0.099	0.007	eGFR	-0.269	-0.276	<0.001
eGFR	-0.276	-0.195	<0.001	LDL cholesterol	3.447	0.235	<0.001
LDL cholesterol	5.186	0.243	<0.001	Male sex	3.347	0.118	0.002
Plasma sodium	0.842	0.080	0.025	Leukocyte telomere length		0.017	0.663
Leukocyte telomere length		0.006	0.877				
**Augmentation index@75 (R square 0.571)**				**L_g10_Pulse wave velocity (R square 0.595)**			
	**B**	**Beta**	**P**		**B**	**Beta**	**P**
(Constant)	9.704			(Constant)	0.409		
Age	0.470	0.473	<0.001	Age	0.004	0.466	<0.001
BMI	-0.288	-0.107	0.002	Low alcohol intake category	-0.012	-0.062	0.025
Male sex	-11.563	-0.498	<0.001	Present smoker	-0.027	-0.095	0.001
Present smoker	3.127	0.089	0.002	Aortic systolic BP	0.001	0.209	<0.001
PWV	0.522	0.089	0.021	Heart rate	0.002	0.178	<0.001
SVRI	0.004	0.186	<0.001	HDL cholesterol	-0.019	-0.086	0.007
LDL cholesterol	0.925	0.077	0.023	Uric acid	0.0002	0.165	<0.001
Leukocyte telomere length	-2.334	-0.065	0.032	Glucose	0.013	0.080	0.010
				Leukocyte telomere length		-0.002	0.936

BP, blood pressure; BMI, body mass index; eGFR, cystatin C and creatinine based CDK-EPI formula for eGFR [59]; LDL, low density lipoprotein; PWV, pulse wave velocity; SVRI, systemic vascular resistance index; HDL, high density lipoprotein.

**Table 5:** Linear regression analyses with backward elimination for the hemodynamic variables including short telomere proportion in the explanatory factors.

**Aortic systolic BP (R square 0.337)**				**Aortic pulse pressure (R square 0.229)**			
	**B**	**Beta**	**P**		**B**	**Beta**	**P**
(Constant)	-24.599			(Constant)	-52.148		
Age	0.278	0.158	0.001	Age	0.296	0.336	<0.001
BMI	0.991	0.209	<0.001	BMI	0.295	0.124	0.003
High alcohol intake category	13.398	0.099	0.007	Male sex	-1.862	-0.090	0.025
eGFR	-0.276	-0.195	<0.001	Low alcohol intake category	1.645	0.075	0.049
LDL cholesterol	5.186	0.243	<0.001	LDL cholesterol	1.548	0.145	0.001
Plasma sodium	0.842	0.080	0.025	Plasma sodium	0.491	0.094	0.018
Short telomere proportion		-0.049	0.196	Short telomere proportion		-0.008	0.851
**Augmentation index@75 (R square 0.568)**				**L_g10_ of Pulse wave velocity (R square 0.595)**			
	**B**	**Beta**	**P**		**B**	**Beta**	**P**
(Constant)	-11.620			(Constant)	0.409		
Age	0.489	0.492	<0.001	Age	0.004	0.466	<0.001
BMI	-0.261	-0.098	0.005	Low alcohol intake category	-0.012	-0.062	0.025
Male sex	-11.413	-0.492	<0.001	Present smoker	-0.027	-0.095	0.001
Present smoker	3.268	0.093	0.001	Aortic systolic BP	0.001	0.209	<0.001
PWV	0.528	0.090	0.020	Heart rate	0.002	0.178	<0.001
SVR	0.004	0.183	<0.001	HDL cholesterol	-0.019	-0.086	0.007
LDL cholesterol	0.934	0.078	0.022	Uric acid	0.0002	0.165	<0.001
Short telomere proportion		0.018	0.542	Glucose	0.013	0.080	0.010
				Short telomere proportion		0.011	0.703

BP, blood pressure; BMI, body mass index; eGFR, cystatin C and creatinine based CDK-EPI formula for eGFR [59]; LDL, low density lipoprotein; PWV, pulse wave velocity; SVR, systemic vascular resistance; HDL, high density lipoprotein.

Mean LTL was not an explanatory factor for aortic systolic BP, aortic diastolic BP, or PWV, in contrast to age, and several cardiovascular risk factors (Table 4). However, mean LTL was a moderate but independent explanatory factor for AIx@75 (beta -0.065, p=0.032; Table 4) and AIx (beta -0.063, p=0.033; Supplementary Table 1). In contrast, STP was not an explanatory factor for any of the hemodynamic variables (Table 5). Finally, neither mean LTL (beta 0.008, p=0.765) nor STP (beta -0.037, p=0.167) were explanatory variables for SVRI.

## DISCUSSION

In this study we examined whether mean LTL or STP were associated with hemodynamic variables in 566 normotensive and never-treated hypertensive volunteers. None of the subjects used medications with direct influences on cardiovascular function. We found multiple significant correlations between mean LTL or STP and hemodynamic variables in univariate analyses. However, in the regression analyses AIx, a marker of wave reflections, was the only hemodynamic variable that was moderately but independently related with mean LTL, while none of the hemodynamic variables were independently related with STP.

The most important task of telomeres is to prevent chromosome heads from attaching to each other and to protect chromosomes from inadequate DNA repair mechanisms [1]. After telomeres reach a critically short stage, the cells enter apoptosis [7]. The enzyme telomerase, found e.g. in stem cells and cancer cells, can maintain telomere length and enable almost limitless number of cell replication cycles [24]. A multitude of extrinsic and intrinsic factors like oxidative stress and inflammation accelerate telomere shortening [7, 25]. In concert with the concept that telomere shortening takes place during aging [6, 7, 26], age was the most important determinant of mean LTL and STP in the linear regression analyses of the present study.

We found that in addition to age, the explanatory variables for mean LTL and STP in regression analyses were BMI and alcohol consumption category, and in the case of STP also male sex. Benetos et al. suggested that for a given chronological age, biological aging in men is more advanced than in women [10]. The finding that male sex was an independent explanatory variable for STP in our study corresponds to this view. A previous meta-analysis found that higher BMI was associated with shorter telomeres, especially in younger individuals [27], while shorter LTL was also found to predict the development of insulin resistance, a condition is closely associated with higher BMI [28]. Moderate alcohol consumption has been suggested to be beneficial for health, and low and moderate alcohol intake might have some protective effect on all-cause and cardiovascular mortality [29]. Moderate beer consumption may even provide some benefit against cardiovascular disease [30]. While a meta-analysis reported that the results concerning alcohol consumption and LTL are unclear [31], a disorder with heavy alcohol use was associated with telomere shortening [32]. In the present study, moderate alcohol consumption category was associated with shorter telomeres, while low alcohol intake category was associated with lower proportion of short telomeres. These findings do not support the view that moderate alcohol consumption would be beneficial for LTL.

In the course of aging, the amounts of elastin and smooth muscle cells decrease in arteries whereas the quantity of collagen increases [33]. These changes result in increased arterial stiffness, manifested as higher PWV, increased wave reflections, and elevated pulse pressure [13, 34]. Another factor contributing to arterial stiffness is atherosclerosis, characterized by inflammation and increased turnover of the intimal and medial cells in the vascular wall [33]. Previously, changes in telomere length have been related with increased arterial stiffness. Among 120 men and 73 women, men but not women with shorter LTL were more likely to have increased PWV and higher pulse pressure [10]. In a population of patients with cardiovascular disease, hypertension, diabetes, and chronic kidney disease, shorter telomeres were associated with increased arterial stiffness [9]. Although these results were adjusted for the use of antihypertensive medications, diabetes, and hyperlipidemia, these factors remain a potential source of confounding. In contrast, Denil et al. found no significant correlations between LTL and the hemodynamic variables PWV, pulse pressure, and left ventricular mass in 2524 subjects of the Asklepios study [19]. Furthermore, longer LTL was even associated with higher left ventricular mass in 334 Flemish participants, a finding that was related to the role of telomere length in determining cardiomyocyte replication [35].

Recently, age- and sex-adjusted LTL, but not muscle telomere length, was found to be shorter in patients with atherosclerotic cardiovascular disease than in controls [36]. Increased LTL attrition due to genetic and environmental factors was considered as the likely explanation for shorter LTL in atherosclerotic cardiovascular disease, and shorter LTL was suggested as the predisposing factor for atherosclerosis [36]. In that study, the mean subject age (67 vs. 58 years), and proportions of women (17% vs. 47%), smokers (present 74% vs. previous 30%), and patients with hypertension (61% vs. 30%), diabetes (26% vs. 13%), and dyslipidemia (38% vs. 8%) were different between the study groups (atherosclerotic cardiovascular disease vs. controls, respectively), and such differences make a potential source of confounding [36].

In the present study, AIx was the only hemodynamic variable with a small independent influence on LTL in the regression analyses. Therefore, our results suggest that the influences of chronological and biological aging in the vasculature are reflected in the magnitude of the AIx. The level of AIx is influenced by arterial stiffness, heart rate, ventricular ejection duration, body height, BP, systemic vascular resistance, and stroke volume [21–23, 37, 38]. From these variables, BP and arterial stiffness are known to increase in the course of aging [23, 34], corresponding to the findings of the present study. The present results also showed that the level of SVRI was directly related with age (r_S_=0.290). In contrast, heart rate, ventricular ejection duration, and stroke volume were not associated with age in our study population.

Aging, obesity and cardiovascular risk factors like smoking and heavy alcohol use impair cardiovascular function and are associated with telomere shortening [6, 7]. Although life style factors have a strong impact on telomere shortening [6], socioeconomic status that is strongly associated with health-related behavior [39], was not associated with LTL in the large West of Scotland Coronary Prevention Study comprising 1542 men [40]. Corresponding to the view of the inheritance of shorter telomeres, LTL was shorter in 45 healthy offspring of subjects with premature coronary artery disease than in 59 offspring from families without such history [41].

The present study was carried out in normotensive and never-treated hypertensive subjects, and the multivariable linear regression analyses were adjusted for several factors that influence cardiovascular function. Our results may be generalized to the Finnish population, as the demographic characteristic of the study participants were similar to the results of a recent population study in our country [42]. The study subjects equally represented both sexes with an age range of 19-72 years, which probably reduced a possible selection among the participants. We chose Southern blot measurements for this study as they are more accurate than quantitative polymerase chain reaction (qPCR) –based assessments, although the latter method has been more widely applied [43]. A methodological advantage of Southern blotting over qPCR was that both mean LTL and STP could be determined. Regardless of mean LTL, short telomeres have a more pronounced influence on cell senescence than long telomeres. Therefore, the inclusion of STP gives valuable additional information about the biological influences of telomeres [3].

If we had applied two different methods for LTL determinations that would have yielded similar associations with hemodynamic variables, the conclusions of the study would be stronger. Two previous studies that compared Southern blotting and qPCR for LTL analyses found correlations exceeding 0.8 between these two methods [44, 45], but in the report by Elbers et al. the correlation was lower 0.52 [43]. Thus, the outcome of the analyses may differ depending on the method of LTL determination. Southern blot measures mean telomere restriction fragment length containing both the canonical and noncanonical components of telomeres, not absolute canonical length [44, 46]. The qPCR technique measures only the canonical component of telomeres, and the outcome of qPCR is normalized to a single gene to provide a mean telomere length for the examined leukocytes [44, 45]. However, the measurement error defined by coefficient of variation is significantly higher for qPCR than for Southern blotting [43, 44]. Aviv et al. concluded that measurement error should be the primary consideration in cross-sectional studies that examine LTL over a wide age range [44], a view that strongly supports the use of Southern blotting in the present study with participant ages ranging 19-72 years. Altogether, the qPCR method is better suited for large genetic epidemiological studies [45, 47]. The other limitations of this study originate from the hemodynamic measurements that were implemented using non-invasive methods, which require mathematic equations and physiological simplifications [48]. However, the present methods have been found to be reliable and repeatable [48–51]. PWV was recorded by the use of the bioimpedance signal and not the tonometric method, which is considered to be the gold standard for estimating arterial stiffness [52]. However, PWV measured using impedance cardiography correlates well with the values measured using either Doppler ultrasound or the tonometric method [22, 53]. Also, PWV measured by the present method independently predicted incident hypertension in 1183 Finnish adults aged 30-45 years [54]. Naturally, the cross-sectional study design of this study does not allow conclusions about causality between LTL and cardiovascular variables.

A significant association between short LTL and increased odds ratio for stroke and myocardial infarction was reported in a meta-analysis [55]. Another meta-analysis comprising 24 studies found ~50% increase in relative risk for coronary heart disease when the shortest versus the longest tertile of LTL were compared [56]. However, epidemiological research has found very low effect sizes for the influences of telomere length on cardiovascular outcomes, and uncertainty exists whether telomere shortening is truly a cause or merely a consequence of cardiovascular disease [20]. In the present study, AIx that is a variable of wave reflection, was the only hemodynamic factor that was moderately but independently related with mean LTL. These findings suggest that the influences of chronological and biological aging are reflected in the magnitude of the AIx.

## MATERIALS AND METHODS

### Study subjects and laboratory analyses

The data originates from the DYNAMIC-study on hemodynamics (clinicaltrials.gov NCT01742702). Announcements subject recruitment were distributed in Tampere University and Tampere University Hospital, and announcements were published in a newspaper. Moreover, beginners in Varala Sport Institute’s long-distance running program were informed about the opportunity to participate in this research, and occupational health care offices around Tampere were informed that their clients could participate. The subjects who were interested contacted directly the research nurse. The present study included 566 subjects (287 men, i.e. 50.7 % of participants), who were screened from altogether 878 subjects (480 men and 398 women) who had participated in the hemodynamic measurements.

All included subjects were without antihypertensive medications or other compounds with potential direct influences on hemodynamics [57]. Altogether 162 (28 %) of the 566 persons used some medications. Seventy-four female subjects used systemic estrogen, progestin, or their combination (for contraception or hormone replacement therapy), 31 participants were on antidepressants or anxiolytics, 13 took antihistamines, 12 medications for asthma, 11 statins, 9 proton pump inhibitors, while 16 euthyroid subjects were on a stable dose of thyroid hormone. Some other medications (small dose acetylsalicylic acid, nonsteroidals, varenicline, pregabalin, sulphasalazine, allopurinol, alendronate) were each used by ≤5 subjects of the study population. One physically well and symptomless subject was taking warfarin because of an anti-phospholipid syndrome.

A physician interviewed and examined all subjects. The interview included smoking habits, weekly alcohol consumption, physical activity, family history of cardiovascular diseases, and subject’s chronic diseases and medications. The exclusion criteria were: use of antihypertensive medications, secondary hypertension [58], diabetes, coronary artery disease, previous myocardial infarction, atherosclerotic vascular disease, cardiac insufficiency, renal disease, cerebrovascular disease or stroke, atrial fibrillation, heart valve disease, and prevalent malignant disease.

Blood and urine samples were taken after ~12 hours of fasting. Plasma cystatin C, uric acid, CRP, glucose, sodium, potassium, creatinine, triglyceride and total, HDL-C and LDL-C were determined by Cobas Integra 700/800 chemistry analyzer (F. Hoffmann-LaRoche Ltd, Basel, Switzerland) or Cobas6000, module c501 (Roche Diagnostics, Basel, Switzerland). To exclude patients with renal diseases, urine dipstick analysis was made using an automated refractometer test (Siemens Clinitec Atlas or Advantus, Siemens Healthcare GmbH, Erlangen, Germany). Cystatin C and creatinine based eGFR was calculated using the CKD-EPI formula [59].

### Experimental protocol

Hemodynamic measurements were performed by research nurses in a quiet, temperature-regulated room. The subjects were instructed not to consume caffeine or nicotine containing products or a heavy meal for at least 4 hours or alcohol for at least 24 hours before the measurements. During the measurements, the subjects lay supine on the recording table with the electrodes for impedance cardiography placed on body surface, the tonometric sensor for pulse wave analysis on radial pulsation of the left wrist, and the oscillometric brachial cuff for BP calibration on the right upper arm [38, 51, 57]. Hemodynamic data was captured continuously for 5 minutes. For statistical analyses the mean values of last 3-minute period were calculated because then the signals were the most stable. The good repeatability and reproducibility of the measurement protocol has been demonstrated [51].

### Pulse wave analysis

BP and pulse wave form were continuously captured from the radial pulsation by a tonometric sensor (Colin BP-508T, Colin Medical Instruments Corp., USA), which was attached to the left wrist using a band. Radial BP was calibrated approximately every 2.5 minutes by a brachial BP measurement from the opposite arm. Aortic BP, aortic pulse pressure, aortic reflection time, AIx (augmented pressure/pulse pressure*100), AIx@75, and pulse pressure amplification (radial pulse pressure/ aortic pulse pressure) were determined with the SphygmoCor software (SpygmoCor PWMx, Atcor medical, Australia) [49].

### Whole-body impedance cardiography

The whole-body impedance cardiography device (CircMon^R^, JR Medical Ltd., Tallinn, Estonia) records changes in body electrical impedance during cardiac cycles. The device was used to determine heart rate, stroke volume, cardiac output, and PWV [48, 53]. Systemic vascular resistance was calculated from the BP signal of the radial tonometric sensor and the cardiac output measured by the Circmon^R^ device by subtracting the assumed central venous pressure (3 mmHg) from mean arterial pressure and dividing it by cardiac output. Cardiac output and systemic vascular resistance were related to body surface and presented as indexes (CI and SVRI respectively). The method and electrode configuration has been previously reported [48, 53].

### Southern blotting

Mean LTL and the proportion of short telomeres (<5 Kb) were measured using the Southern blot technique as previously described in detail [60]. The interassay coefficient of variation for LTL determination was 3.7% when calculated from an internal control DNA sample in 96 assays.

### Statistics

The data were analyzed by SPSS version 25.0 (IBM SPSS Statistics, Armonk, NY, USA). Spearman correlations coefficients (r_S_) between LTL and proportion of short telomeres and other variables were calculated for the scatter plots. Alcohol intake was analyzed as series of discrete variables that were given a score of either 0 or 1; the cut-points for women were 0, 1-7, 8-14, and above 15 standard 12-gram doses of alcohol per week; for men 0, 1-14, 15-24, and above 25 doses of alcohol per week, according to the Finnish Guidelines [61]. Information about alcohol intake was missing from 10 men and 11 women and this was replaced by the mean value of each gender in the statistical analyses. As smoking of one cigarette per day already carries the risk of developing coronary heart disease that is about half of that for people who smoke 20 cigarettes per day [62], smoking status was categorized as two discrete variables that were given a score of either 0 or 1: this categorization contained the information whether the person was a present smoker, previous smoker or never smoker. Power calculations indicated that in order to detect a 2 percentage point difference in the magnitude of the augmentation index or AIx@75 (two-tailed alpha level 0.05, 80 % power, standard deviation of 11 percentage points), the required study population size was at least 237 subjects.

Linear regression analyses with backward elimination was applied to evaluate the associations between LTL and proportion of short telomeres with age, sex, BMI, smoking status, categorized alcohol intake, plasma CRP, sodium, potassium, uric acid, LDL-C, HDL-C, triglycerides, creatinine and cystatin C based eGFR [59], and aortic systolic and diastolic BP, AIx and AIx@75, SVRI, and PWV. The skewed distributions of PWV and triglycerides were corrected by Lg_10_-transformation for the statistics. The variables that were included in the linear regression analyses correlated with *P* value <0.1 in univariate analyses with the variable of interest. P-values <0.05 were considered statistically significant.

## Supplementary Material

Supplementary Figure 1

Supplementary Table 1
