# Supplementary material for: Leukocyte telomere length is inversely associated with arterial wave reflection in 566 normotensive and never-treated hypertensive subjects
Source: Aging (Albany NY). 2020 Jun 23;12(12):12376–92. doi: 10.18632/aging.103459 (PMC7343461; doi:10.18632/aging.103459)
Supplement: Supplementary Figure 1 [file aging-12-103459-s001..pdf]

## SUPPLEMENTARY FIGURE

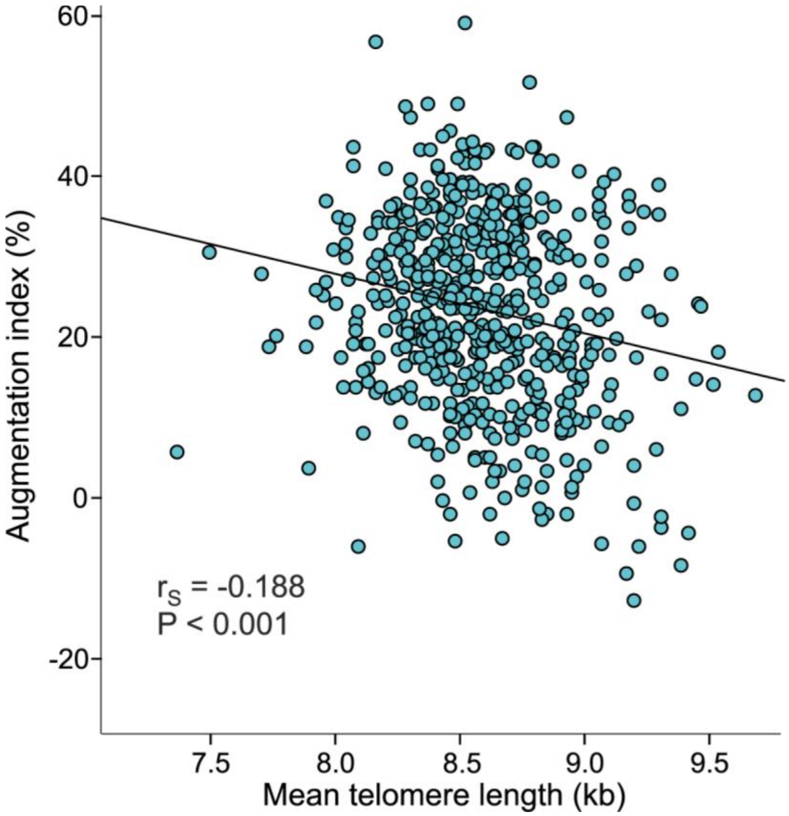


**Supplementary Figure 1. Scatter plots and Spearman correlations (rS) between mean leukocyte telomere length and augmentation index.**
