# Supplementary material for: Leukocyte telomere length is inversely associated with arterial wave reflection in 566 normotensive and never-treated hypertensive subjects
Source: Aging (Albany NY). 2020 Jun 23;12(12):12376–92. doi: 10.18632/aging.103459 (PMC7343461; doi:10.18632/aging.103459)
Supplement: Supplementary Table 1 [file aging-12-103459-s002..pdf]

SUPPLEMENTARY TABLE

Supplementary Table 1. Linear regression analyses with backward elimination of the explanatory factors for the magnitude of augmentation index.

	Augmentation index (R square 0.604)		
	**B**	**Beta**	**P**
(Constant)	-4.175		
Age	0.532	0.530	<0.001
Male sex	-11.747	-0.502	<0.001
SVRI	0.007	0.378	<0.001
Stroke index	0.270	0.164	<0.001
BMI	-0.339	-0.126	0.002
Uric acid	0.014	0.091	0.017
Heart rate	-0.098	-0.079	0.029
Present smoker	2.661	0.075	0.007
Leukocyte telomere length	-2.253	-0.063	0.033

SVRI, systemic vascular resistance index; BMI, body mass index.
